# Draft genome assemblies of two *Beauveria bassiana* strains from Taiwan using the hybrid assembly approach

**DOI:** 10.1128/mra.01151-25

**Published:** 2025-11-26

**Authors:** Kusum Mushyakhwo, Yu-Shin Nai

**Affiliations:** 1 International Doctoral Program in Agriculture, National Chung Hsing University 34916, Taichung, Taiwan; 2 Department of Entomology, National Chung Hsing University 34916, Taichung, Taiwan; Rochester Institute of Technology, Rochester, New York, USA

**Keywords:** *Beauveria bassiana*, hybrid assembly, whole-genome sequencing

## Abstract

We report the draft genome sequences of two *Beauveria bassiana* strains, Bb-NCHU-143 and Bb-NCHU-153, isolated from mycosed coleopteran (beetles) in Taiwan. Genome assemblies were generated using a hybrid genome assembly approach. The draft genomes of Bb-NCHU-143 and Bb-NCHU-153 are 34.21 Mb (7,994 genes) and 34.20 Mb (7,151 genes), respectively.

## ANNOUNCEMENT

*Beauveria bassiana* is a widely studied entomopathogenic fungus (EPF), well known for its effectiveness as a biocontrol agent against a variety of insect pests. In this study, we report the hybrid genome assemblies of two indigenous *B. bassiana* strains, Bb-NCHU-143 and Bb-NCHU-153, which exhibit high pathogenicity against the agricultural pest, fall army worm (*Spodoptera frugiperda*) (1).

The EPF strains, Bb-NCHU-143 and Bb-NCHU-153, were isolated from cadavers of coffee berry borer (*Hypothenemus hampei*) from Chiayi, Taiwan and red palm weevil (*Rhynchophorus ferrugineus*) from Changhua, Taiwan, respectively. Isolation, purification, and identification of the strains have been previously reported (1–3). The strains were stored at −80°C in 20% glycerol at the Insect Pathology and Genomics Laboratory, National Chung Hsing University (Taichung, Taiwan). The EPF strains were recovered from −80°C and cultured on quarter-strength Sabouraud dextrose agar (HiMedia Co.) at 25°C for 10 days in darkness for genomic DNA extraction.

Fungal genomic DNA was extracted using a Yeast Genomic DNA Kit (Geneaid, Taiwan), following the manufacturer’s protocol. The extracted DNA was subjected to both Illumina and Oxford Nanopore Technologies (ONT) sequencing. For Illumina sequencing, a library was constructed using NEBNext Ultra II DNA Library Prep Kit for Illumina (NEB, USA) and sequenced on an Illumina HiSeq platform with paired-end (PE) reads of 2 × 150 bp (Macrogen, Korea). Quality trimming and adapter removal of the NGS reads were performed using Trimmomatic v0.39 (4). For ONT sequencing, the library was prepared using the Ligation Sequencing Kit SQK-LSK109 (ONT, Cambridge, UK) and sequenced on an R9.4.1 SpotON flow cell using a MinION device operated with ONT MinKNOW software version 22.05.5. ONT sequencing reads were trimmed using Porechop v0.2.4 (5) (https://github.com/rrwick/Porechop) to remove adapters. The raw Illumina sequencing data for strain Bb-NCHU-143 consisted of 42,561,883 reads, with 35,927,457 reads retained after quality filtering. For strain Bb-NCHU-153, a total of 39,237,588 reads were generated, of which 32,371,398 high-quality reads remained after filtering. ONT sequencing followed by Guppy basecalling v6.1.7 (ONT community site: https://community.nanoporetech.com) (6), produced 468,422 reads (2,989,362,299 bases) and 81,298 reads (637,332,762 bases) for Bb-NCHU-143 and Bb-NCHU-153, respectively. After adapter trimming, 468,140 reads (2,937,027,379 bases) and 81,304 reads (633,983,475 bases) for Bb-NCHU-143 and Bb-NCHU-153, respectively, were used for hybrid genome assembly.

Genome assembly was performed using MaSuRCA v4.1.0 (7), integrating both Illumina and ONT sequencing reads. Default parameters were used, except: LHE_COVERAGE = 15, JF_SIZE = 1000000000, CA_PARAMETERS = cgwErrorRate = 0.15 ovlMinLen = 50. Assembly quality was evaluated using QUAST v5.2.0 (8), and completeness was evaluated using BUSCO v5.8.2 (9) against the fungal_odb10 database. The fungal genome was annotated using BRAKER (10–12), incorporating RNA-seq-based gene prediction. Public RNA-seq data (SRR3105334; PRJNA309019) from NCBI were aligned to the assembly with HISAT2 v2.2.1 (13). Transfer RNA (tRNA) genes were predicted using tRNAscan-SE v2.0 (14). Secondary metabolite gene clusters were identified using the antiSMASH web server (15). Transposable element (TE) regions were predicted with RepeatModeler2 (16) and annotated using RepeatMasker (17). The information on genome assembly and analysis is summarized in Table 1. Default parameters were used except where otherwise noted.

**TABLE 1:** Genome features of *Beauveria bassiana* strains assembly and annotation

Assembled genome	Bb-NCHU-143	Bb-NCHU-153
Number of contigs	11	13
Largest contig (bp)	7,146,430	7,946,388
Total length (bp)	34,214,673	34,200,021
Coverage	387.59×	290.12×
GC (%)	50.69	50.52
*N*_50_ (bp)	5,547,362	5,644,419
Complete BUSCO marker genes (%)	99.60%	99.70%
Fragmented BUSCO marker genes (%)	0.10%	0.00%
Missing BUSCO marker genes (%)	0.30%	0.30%
Total protein-coding genes	7,994	7,151
Gene density (gene/Mb)	233.64	209.09
Number of exons	20,901	19,009
Number of exons per gene	2.62	2.66
Number of tRNA	127	200
Number of secondary metabolites	38	41
Repeat sequence density (%)	5.94	6.36
TE density	6.49%	6.83%

## Data Availability

The *Beauveria bassiana* genome project, including the raw Illumina and Nanopore data, has been deposited in NCBI under BioProject accession number PRJNA1237937. This whole-genome shotgun project has been deposited at DDBJ/ENA/GenBank under the accession numbers JBPXMI000000000 (*Beauveria bassiana*-NCHU-143) and JBPYGC000000000 (*Beauveria bassiana*-NCHU-153). The versions described in this paper are version JBPXMI010000000 (Bb-NCHU-143) and JBPYGC010000000 (Bb-NCHU-153). The raw Illumina sequencing reads have been deposited under accession numbers SRR32764875 (Bb-NCHU-143) and SRR32764876 (Bb-NCHU-153). The Nanopore sequencing data were deposited under accession numbers SRR32764873 (Bb-NCHU-143) and SRR32764874 (Bb-NCHU-153).
